# Effect of Substrate Grain Size on Structural and Corrosion Properties of Electrodeposited Nickel Layer Protected with Self-Assembled Film of Stearic Acid

**DOI:** 10.3390/ma13092052

**Published:** 2020-04-28

**Authors:** Amir Hossein Noorbakhsh Nezhad, Ehsan Rahimi, Reza Arefinia, Ali Davoodi, Saman Hosseinpour

**Affiliations:** 1 Materials and Metallurgical Engineering Department, Faculty of Engineering, Ferdowsi University of Mashhad (FUM), Mashhad 9177948974, Iran; 2 Department of Engineering and Architecture, University of Udine, Via Cotonificio 108, 33100 Udine, Italy; 3 Chemical Engineering Department, Faculty of Engineering, Ferdowsi University of Mashhad (FUM), Mashhad 9177948974, Iran; 4 John A. Paulson School of Engineering and Applied Sciences, Harvard University, Cambridge, MA 02138, USA; 5 Institute of Particle Technology (LFG), Friedrich-Alexander-University Erlangen-Nürnberg (FAU), Cauerstrasse 4, 91058 Erlangen, Germany

**Keywords:** grain-size effect, electrodeposition, nickel coating, XRD, self-assembled layers

## Abstract

In the present study, the impact of copper substrate grain size on the structure of the succeeding electrodeposited nickel film and its consequent corrosion resistance in 3.5% NaCl medium were evaluated before and after functionalization with stearic acid. Nickel layers were electrodeposited on two different copper sheets with average grain size of 12 and 25 µm, followed by deposition of stearic acid film through self-assembly. X-ray diffraction analysis of the electrodeposited nickel films revealed that the deposition of nickel film on the Cu substrate with small (12 µm) and large (25 µm) grains is predominantly governed by growth in the (220) and (111) planes, respectively. Both electrodeposited films initially exhibited a hydrophilic nature, with water-contact angles of 56° and <10°, respectively. After functionalization with stearic acid, superhydrophobic films with contact angles of ~150° were obtained on both samples. In a 3.5% NaCl medium, the corrosion resistance of the nickel layer electrodeposited on the copper substrate with 25 µm grains was three times greater than that deposited on the copper substrate with 12 µm grains. After functionalization, the corrosion resistance of both films was greatly improved in both short and long immersion times in 3.5% NaCl medium.

## 1. Introduction

Hierarchical micro-/nanostructured materials with various shapes and properties are widely used in different applications like as optical materials [1], low adhesive surfaces [2], abrasion-resistant surfaces, anti-icing films [3,4,5,6], anticorrosion coatings [7,8,9], and in the fabrication of superhydrophobic surfaces [10,11]. The superhydrophobicity phenomenon was first observed in lotus leaves in nature [12,13], and it is frequently used to describe surface properties with a water contact angle (CA) larger than 150° and sliding angle less than 10° [14,15]. The fabrication of hierarchical structures for tuning surface hydrophobicity has been demonstrated for various metals, including aluminum [16], zinc [17], and nickel [8], among which nickel is especially attractive because of its high corrosion resistance, attractive visual appearance, and reasonable hardness [18]. Numerous methods, including electrodeposition [7,10], chemical-vapor deposition [19], etching [20], electrospinning [21], and sol–gel [1] have been used so far for the fabrication of nickel films that are decorated with micro-/nanostructures. In this regard, electrodeposition is very attractive in comparison with other methods due to its simplicity, ease of control, minimal required material preparation, and relatively cheap equipment [9]. In the electrodeposition process, metal ions from anodes preferably nucleate and grow on the lattice defects of cathode surface [18,22] where the screw-dislocation-driven growth mechanism is dominant for 2D and 3D growth during electrocrystallization [23,24]. With the gradual increase of supersaturation in the system, the crystal-growth mode progressively changes from screw-dislocation- to layer-by-layer- and then to dendritic-growth mechanisms [18,23,25]. The applied current density also controls the crystal-growth mechanism and has direct correlation with supersaturation in the system [25]. Apart from electrodeposition parameters, the substrate’s microstructure, especially the presence of high-energy (active) sites on edges, corners, kinks, and screw dislocation also effectively influence the shape, size, and morphology distribution of the micro-/nanostructures that are formed during electrocrystallization [26]. Therefore, many studies were performed to evaluate the effect of substrate microstructure on the hierarchical structure of the electrodeposited film, and consequently on properties, such as magnetic [27,28] and tensile properties [29], and its catalytic activity [30]. Changes in substrate-surface morphology and consequently the structure of the electrodeposited films were expected to affect the overall corrosion resistance of nickel layers [31,32].

Copper is commonly used as the substrate for the electrodeposition of metallic layers, especially in cases where the combination is desired of heat or the charge-transfer capabilities of copper and the decorative features of top layer Ni coating. Cold-worked substrates such as copper often exhibit residual lattice strain and highly reactive atoms along slip edges and dislocations defects [18]. It is thus expected that lattice defects in a copper substrate with less stable crystalline conditions effectively control the kinetics of crystal growth and the specific directions of electrocrystallization during nickel electrodeposition. To elucidate such effects, in this study we assess the influence of copper-substrate-grain size as a result of different degrees of cold work on the micro-/nanostructure, hydrophobicity, and corrosion properties of an electrodeposited nickel film in a saline solution. For this purpose, we electrodeposited nickel films with hierarchical micro-/nanostructures on copper substrates with different grain sizes, and evaluated the corrosion resistance of the electrodeposited films in 3.5% NaCl. We further functionalized the micro-/nanostructured electrodeposited nickel films with stearic acid (SA) molecules to lower surface energy and improve its corrosion resistance. SA application for the improvement of corrosion resistance of metallic substrates [33], and the way its deposition can reduce surface free energy [16] were experimentally demonstrated earlier. The low surface free energy of SA molecules stems from the arrangement of aliphatic hydrocarbons in its backbone and its terminal methyl group. Deposition of SA molecules on electrodeposited layers occurs via a self-assembly mechanism, in which adsorbate assembly takes place spontaneously without the need for external forces. The self-assembled molecules can deposit as mono- or multilayers, and can strongly or weakly interact with the substrate, depending on the anchoring groups in the adsorbates and their interaction affinity with the substrate [34]. Application of the self-assembled layers of organic molecules for the protection of metallic substrates against corrosion and oxidation has been the subject of numerous studies [35].

We used the texture-coefficient (TC) parameter, extracted from X-ray diffraction (XRD), to determine the preferential growth direction of the nickel films on different copper substrates. We also used CA measurement to assess the wettability of the fabricated Ni films before and after functionalization, and after exposure to a corrosive saline medium. The microstructure of the electrodeposited nickel layers was investigated with scanning electron microscopy (SEM). We then assessed the resistance of the electrodeposited nickel film against corrosion in a corrosive medium using electrochemical impedance spectroscopy (EIS) and potentiodynamic polarization (PDP). To the best of our knowledge, studies directly correlating substrate-grain size to the microstructure and physiochemical properties of electrodeposited films are scarce; hence, this study puts forward new insights on the fabrication of coatings with improved corrosion resistance.

## 2. Materials and Methods

Continuously cast, hot-rolled, and then soft-annealed pure copper sheets were cold-rolled at two different levels to form different grain sizes (12 and 25 micrometers as average grain diameter, D12 and D25). The ASTM E-112 standard [36] planimetric method was used in determining average grain diameter. Samples were cut into pieces of 1 cm in diameter and used as cathodes during electrodeposition. Prior to electrodeposition, copper samples were ground using successive grades of SiC paper up to a grade of 3000 and then polished with alumina slurry, followed by a rinsing step in deionized water. The polished samples were electropolished at 20 mA·cm^−2^ for 1 min in a solution containing 50 g·L^−1^ Na_2_CO_3_, 10 g·L^−1^ KOH, dipped in a 10 wt % HCl solution for 30 s, and washed using deionized water. A digital-camera-assisted optical microscope was used to visualize the microstructure and different grain sizes of the copper (D12 and D25) substrates. SEM (LEO 1450 VP, Zeiss, Oberkochen, Germany) and XRD (Explorer, GNR, Novara, Italy) were used to assess the microstructure and determine the crystalline structure of the electrodeposited layers.

XRD cathodes (either D12 or D25) were immersed in an electrolyte containing 200 g·L^−1^ NiCl_2_·6H_2_O, 30 g·L^−1^ NH_4_Cl, and 120 g·L^−1^ H_3_BO_3_ for electrodeposition where a nickel ingot with 99.5% purity (40 mm × 40 mm × 1 mm) was used as an anode. The electrolyte was constantly stirred, and its temperature was kept at 60 ± 1 °C. To produce a hierarchical micro-/nanostructured nickel film on the copper substrates, two current levels were employed in two steps during electrodeposition; current density of 20 mA·cm^−2^ for 8 min, followed by current density of 50 mA·cm^−2^ for 1 min. To modify sample hydrophobicity, the electrodeposited Ni films were functionalized by SA molecules for 10 min in a 6 mM·L^−1^ SA solution in ethanol. CA measurements were performed with 4 μL water droplet at ambient temperature using an optical contact-angle meter (Adeeco static/dynamic, Tehran, Iran). ImageJ software (Version 1.151) was used to analyze the CA results. For corrosion-resistance evaluation, electrochemical-impedance-spectroscopy (EIS) measurements were performed with a potential amplitude of ±10 mV at a frequency range of 100–0.01 Hz in a 3.5% NaCl electrolyte using a three-electrode setup (Autolab, PGSTAT 302N, Utrecht, The Netherlands). Potentiodynamic-polarization (PDP) measurements were also performed from the −150 mV cathodic region to the ca +200 mV anodic region with a scan rate of 1 mV·s^−1^. Pt mesh and saturated calomel electrodes (SCEs) were used in the electrochemical measurements as counter and reference electrodes, respectively.

## 3. Results

### 3.1. Surface Microstructure

**Figure 1:**
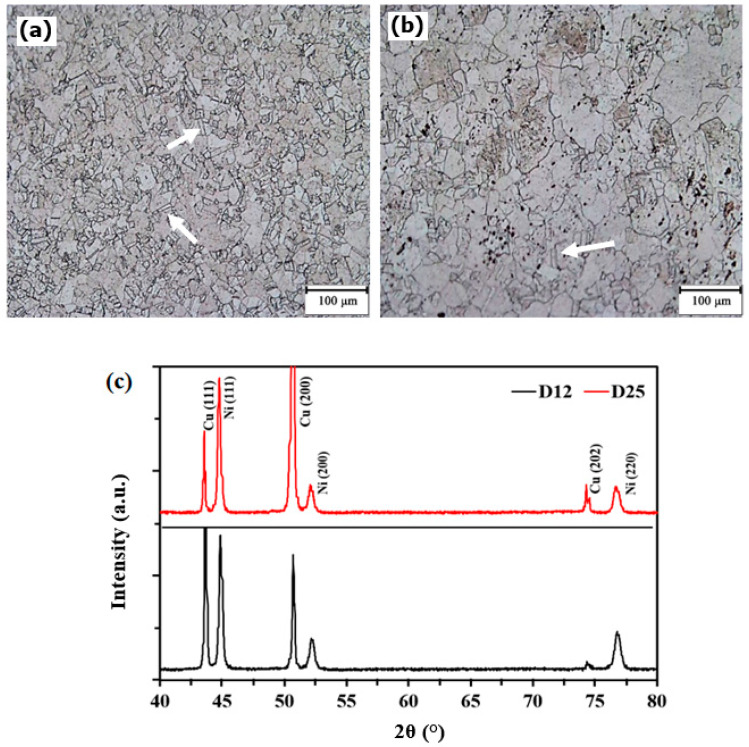
Microstructures of copper substrate with different grain sizes: (**a**) D12 (12 µm average grain size) and (**b**) D25 (25 µm average grain size). Some twins marked with white arrows in SEM images. (**c**) X-ray diffraction patterns of nickel electrodeposited film on D12 and D25 copper substrates.

Figure 1a,b represents the optical microstructure of D12 and D25 that were used as substrates showing their different grain sizes. Sample D12, compared to Sample D25, showed smaller grain sizes, a larger number of grains, and higher density of twins with different sizes and plane directions. According to Hull et al. [37], samples with smaller grains and larger fractions of grain boundaries exhibit greater amounts of crystalline defects, such as subgrain boundaries and dislocations, comparing their larger grains counterparts. Consequently, samples with higher density of crystalline defects better accommodate nucleation and growth sites for electrochemical deposition [18]. A larger density of grain boundaries and high-energy lattice defects (e.g., dislocations, edges, and kinks) on substrates with smaller grain size provides predominant sites for crystal growth, especially based on the screw-dislocation-growth mechanism [18]. Therefore, it could be expected that the Ni electrodeposition process on the D12 substrate led to the formation of an Ni layer with different grain sizes compared to that electrodeposited on the D25 substrate. To assess whether the change of the grain size of the copper substrate affected the crystalline structure of the electrodeposited film, we performed XRD measurements on the electrodeposited Ni on Samples D12 and D25. The thickness of the electrodeposited nickel films on both samples was measured with combined SEM cross-section and elemental-line-profile analyses using energy-dispersive spectroscopy as described elsewhere [8]. The obtained film thickness for the electrodeposited Ni layers in this study was approximately 4–6 µm, consistent with our previous studies [37]. Therefore, the XRD patterns of the nickel films on the D12 and D25 samples (Figure 1c) revealed not only the crystalline structure of the electrodeposited nickel top film, but also different plane directions of the underlying crystalline copper substrate.

Comparing XRD spectra in the above figure, some differences were observed between the structure of the Ni film deposited on Samples D12 and D25. The degree of preferred orientation of particular crystal plane of a polycrystalline nickel film can be determined using texture coefficient (TC) parameter for a specific (*hkl*) plane, as shown by the following equation [6]:

(1) $$TC\;\left(hkl\right)=\frac{I_{hkl\;\left(c\right)}/I_{hkl\;\left(s\right)}}{\frac{1}{n}\sum \;I_{hkl\;\left(c\right)}/I_{hkl\;\left(s\right)}}$$

where *I_hkl(c)_* is the diffraction-peak intensity for the crystalline electrodeposited nickel film, *I_hkl(s)_* is the diffraction peak intensity of the standard nickel powder (as the random state), and *n* is the number of the considered XRD peaks. By changing the copper substrate from D12 to D25, TC (111) and TC (200) increased from 1.04 to 1.24 and from 0.56 to 0.61, respectively, whereas TC (220) decreased from 1.39 to 1.15. In fact, when D12 was used as the substrate, the preferred growth of the electrodeposited nickel film was in the (220) direction, while with the D25 as the substrate, growth was preferential in the (111) direction. These observations revealed direct correlation between the crystalline structure of the electrodeposited nickel film and the substrate microstructure.

### 3.2. Surface Characterization

#### 3.2.1. SEM Investigations

**Figure 2:**
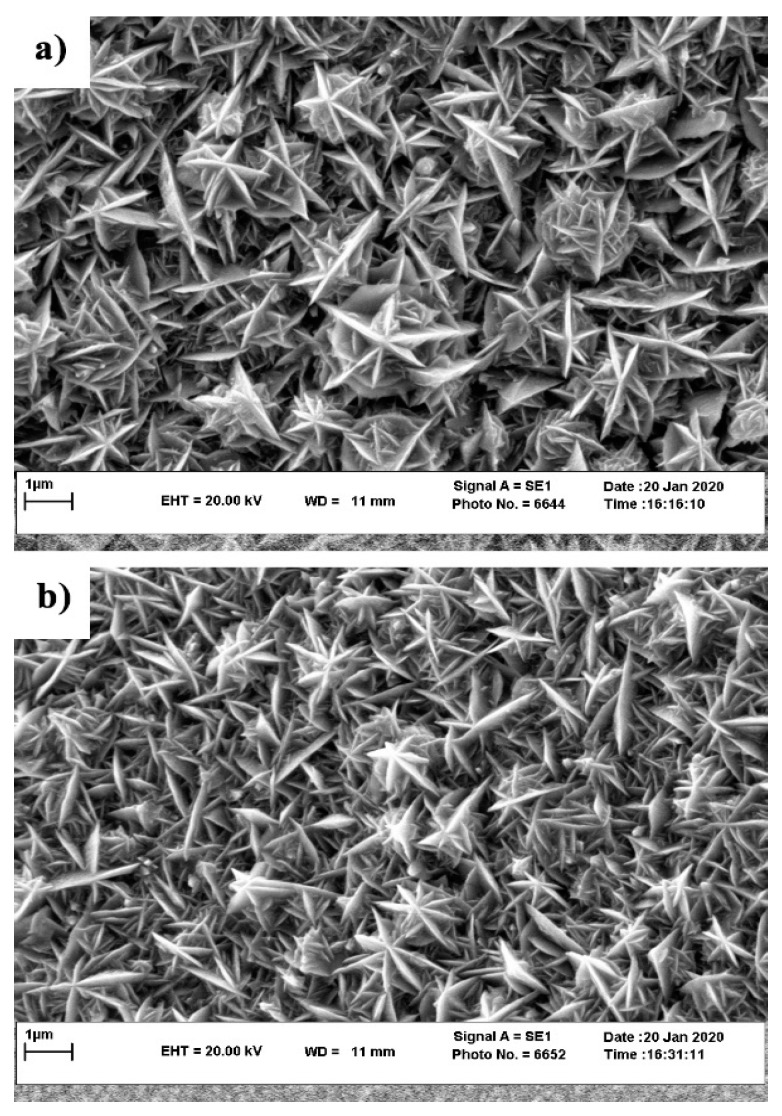
SEM surface morphology of the micro-/nanostructured Ni film on Samples (**a**) D12 and (**b**) D25.

To visualize the effect of substrate-grain size on the micro-/nanostructure of the electrodeposited nickel film, SEM images were obtained on the nickel films deposited on Samples D12 and D25, as shown in Figure 2a,b, respectively. These SEM micrographs clearly show the hierarchical structure of the nickel crystals with their characteristic starlike structure on both substrates. As previously mentioned, Sample D12 provided more available nucleation and growth locations for the Ni film during electrodeposition when compared to Sample D25. Therefore, in the course of electrodeposition, the fusion of neighboring fine Ni grains resulted in the grain coarsening of the Ni film on the D12 copper substrate. Therefore, as shown in Figure 2, the size of starlike features in the film deposited on Sample D12 was slightly larger than that of the film deposited on Sample D25. After functionalization with SA, the surface morphology of the electrodeposited films was unchanged (not shown here), as the thickness of the SA layer is way smaller than the size of features observed in the SEM micrographs.

#### 3.2.2. Surface Hydrophobicity

**Figure 3:**
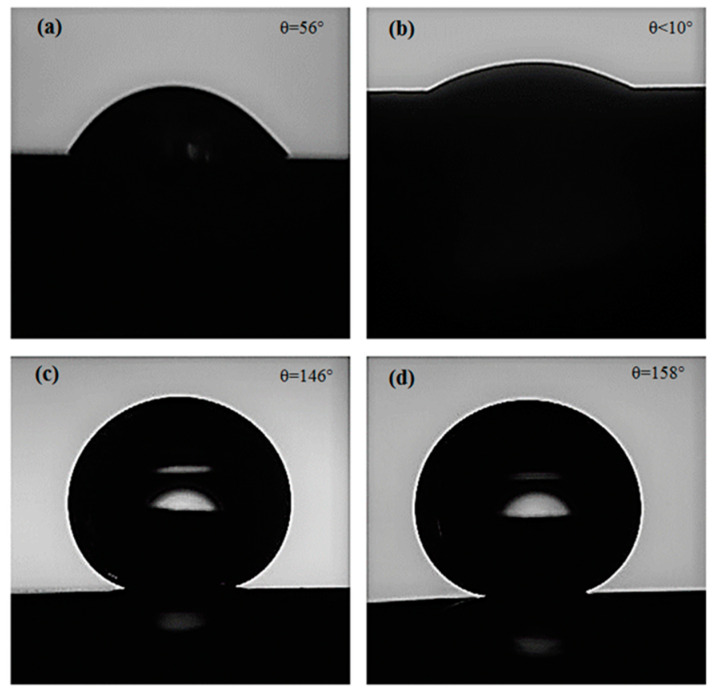
Water droplets during contact-angle measurement on (**a**) D12, (**b**) D25, (**c**) functionalized D12, and (**d**) functionalized D25.

Several factors, such as surface microstructure, surface energy, and surface-oxide growth affect the interactions between an electrode (e.g., electrodeposited Ni film in this case) and an electrolyte. To evaluate the effect of substrate microstructure (i.e., grain size) on the wettability of the electrodeposited Ni films before and after functionalization, we performed water static CA measurements. As can be seen from the CA results in Figure 3a,b, the electrodeposited Ni films on Samples D12 and D25 showed a hydrophilic nature with CA values θ = 56° and θ < 10°, respectively. The lower CA of the electrodeposited Ni film on Sample D25 compared to that on Sample D12 can be explained by the Wenzel model [38] that correlates a decrease in CA to an increase in surface roughness. Nevertheless, since CA measurements were performed in the open laboratory air, the effect of adventurous hydrocarbons on increasing surface hydrophobicity cannot be neglected. In contrast to hydrophilic Ni films before functionalization, functionalized Ni films exhibited a superhydrophobic nature (Figure 3c,d), with their CA close to 150°. As reported earlier [33], adsorption of mono- or multilayer SA molecules on a flat substrate can increase its CA to 100°−110°. If deposited as a single layer, a well-ordered all-trans monolayer of SA molecules exposes the SA hydrophobic terminal methyl group to the water droplet, resulting in high CA. If defects are introduced in the structure of the SA monolayer (also known as gauche defects), the CA decreases, as backbone methylene groups are less hydrophobic than the terminal methyl group. In contrast, when multilayer SA is deposited on a flat substrate, overall CA is determined by all the functional groups of SA protruding the air. Similarly, when a multilayer SA film is formed on rough surfaces, a range of surface hydrophobicity (i.e., contact-angle values) can be expected. CA values observed on functionalized electrodeposited Ni films (i.e., results in Figure 3c,d) were almost 50% higher than the values observed for the SA mono-/multilayers adsorbed on flat surfaces. According to Cassie–Baxter theory [39], the presence of low-surface-energy substances (in this case, SA molecules) on hierarchical micro-/nanostructures results in the entrapment of air pockets between water droplet and surface. The formation of such air pockets can explain the superhydrophobicity that was obtained through functionalization of the electrodeposited Ni films on Samples D12 and D25.

**Figure 4:**
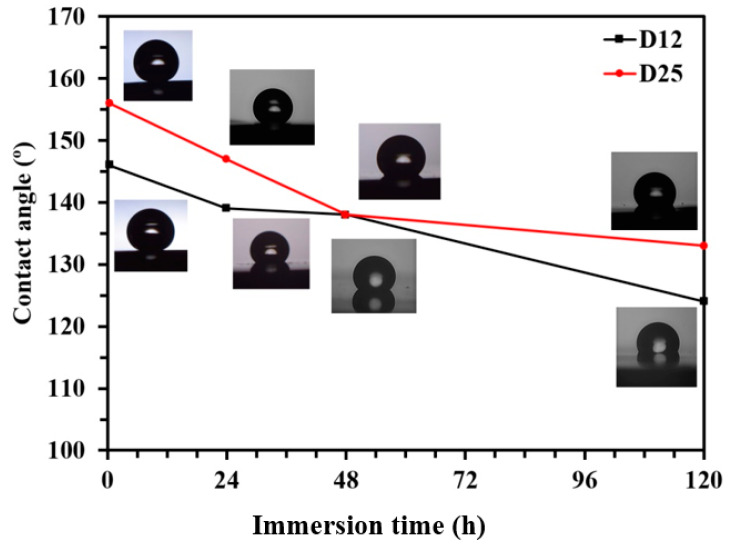
Changes of water contact angle (CA) of functionalized D12 and D25 surfaces with immersion time in 3.5 wt % NaCl solution. Lines connecting symbols (experiment data) are guides; CA values are the average of at least three measurements with <2% standard deviation.

To evaluate the long-term stability of the functionalized electrodeposited Ni films on D12 and D25, the samples were exposed to a 3.5 wt % NaCl solution up to 5 days, and their CA was evaluated at different time intervals. As depicted in Figure 4, the CA of both samples gradually declined upon exposure to 3.5 wt % NaCl solution (by almost 15° after 5 days). Penetration of corrosive Cl^−^ ions through the SA layers, and corrosion/oxidation of the Ni substrate caused the increased concentration of gauche defects in the structure of the adsorbed SA molecules, thus resulting in the reduction of CA values. Oxidation and corrosion of Ni film underneath the SA layer could also result in the further formation of defects in the structure of the SA film. Similar deterioration of the organization of aliphatic organic molecules as a consequence of the oxidation and corrosion of the metallic substrate was demonstrated earlier [40,41,42] for self-assembled monolayers of octadecanethiol and octadecaneselenol on copper. From the high CA values observed on functionalized electrodeposited Ni films on Samples D12 and D25, it could be expected that the functionalized samples exhibited better corrosion-resistance properties compared to their nonfunctionalized counterpart when exposed to a corrosive medium. However, as we recently reported, the CA alone cannot be considered as a measure for the corrosion-protection efficiency of a hybrid substrate [8]. As demonstrated in Figure 4, the CAs of the functionalized and nonfunctionalized samples are shown to be very different. Hence it was expected that the corrosion resistance of these samples in aqueous media also varied to a large extent. To assess the effect of the copper substrate microstructure on the corrosion resistance of the electrodeposited Ni films before and after functionalization, we performed additional electrochemical measurements with potentiodynamic polarization (PDP) and electrochemical impedance spectroscopy (EIS).

### 3.3. Electrochemical Analysis and Corrosion-Resistance Assessment of Electrodeposited Layers

**Figure 5:**
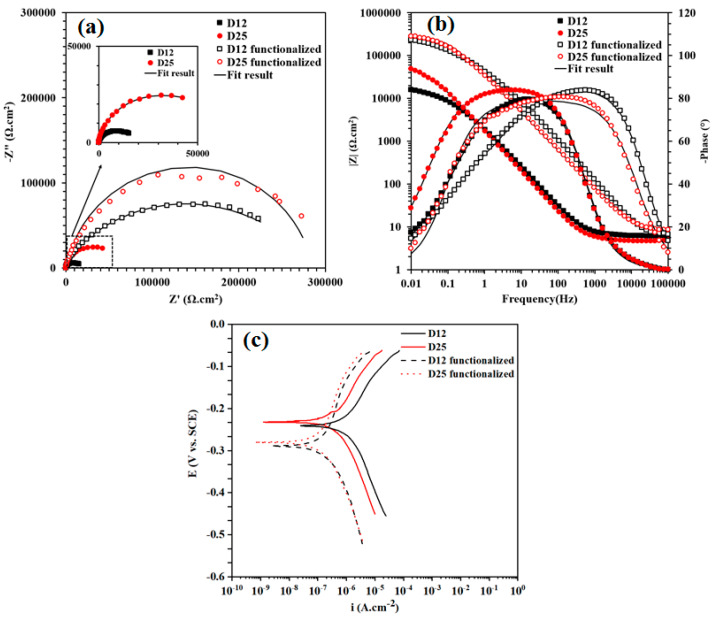
Electrochemical-impedance-spectroscopy (EIS) results for D12, D25, functionalized D12, and functionalized D25 samples exposed to 3.5% NaCl medium at 25 ± 1 °C. (**a**) Nyquist and (**b**) Bode (impedance modulus and phase-angle) representation. (**c**) Potentiodynamic-polarization curves of D12, D25, functionalized D12, and functionalized D25 in 3.5 wt % NaCl solution. Inset in (**a**), zoomed-in representation of area marked with dashed box.

**Figure 6:**
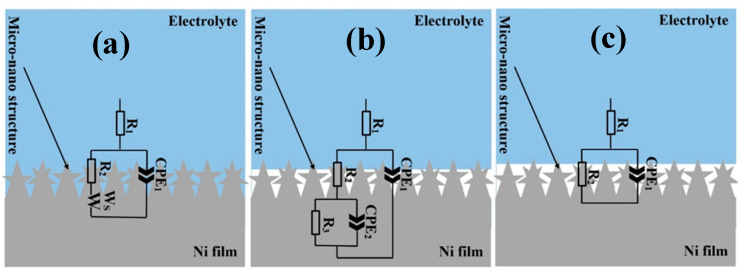
Equivalent circuits models superimposed on schematic representations of (**a**) Samples D12 and D25 before functionalization, (**b**) functionalized D12, and (**c**) functionalized D25 in solution. (**a**–**c**) Micro- and nanostructures schematically presented out of scale.

Results of EIS measurements on the electrodeposited Ni films on Samples D12 and D25 before and after functionalization are provided in Figure 5a,b in Nyquist and Bode representations, respectively. As is observed in Figure 5a, copper substrates with different grain sizes affected the overall corrosion resistance of the electrodeposited Ni films. Before and after surface functionalization, the electrodeposited Ni films on Sample D25 exhibited better corrosion-resistance performance compared to those on Sample D12. To quantitatively evaluate EIS data, we fit EIS spectra using equivalent circuit models as those shown in Figure 6. In this figure, the equivalent circuit models were superimposed on schematic representations of sample-surface constituents, and the obtained corresponding fitting parameters are provided in Table 1. The equivalent circuit models were chosen on the basis of the presence of different surface constituents (e.g., micro-/nanostructured surface, oxide layer, SA molecules, and air pockets) and in accordance with our previous studies on similar systems [8]. Surfaces of the functionalized samples are considered as dynamic systems and undergo changes after immersion in corrosive electrolytes. Therefore, different equivalent circuit models were used to fit the EIS results (Figure 5a,b). As schematically shown in Figure 6a–c, the equivalent circuit models for fitting EIS data consisted of a constant phase element (CPE), parallel-connected with a resistance element, and connected in series to a Warburg short element (*W_s_*) before sample functionalization. The *CPE* parallel-connected with the resistance element is representative of the surface micro-/nanostructure, and *W_s_* is representative of the chlorine ion’s penetration into the surface grooves. For *n* = 1, *CPE* had a unit of capacitance (F). Parameters *C* (capacitance), and *CPE* factors (*Q* and *n*) in Table 1 were calculated using Equations (2) and (3), where *Q* had a unit of Ω·cm^−2^·S^−*n*^.

(2) $$C=\frac{{\left(Q_{C}\times R\right)}^{\left(\frac{1}{n}\right)}}{R}$$

(3) $$CPE=\frac{1}{Q_{C}{\left(j\omega \right)}^{n}}$$

where *Q* is the CPE constant that nominally equals to the pure capacitance of the system for *n* = 1; *j*^2^ = −1; ω is the angular frequency (rad/s); and the value of *n* ranged between 0 and 1. *W_s_* was thus defined as Equation (4), where *R_Ws_* is the short-range Warburg coefficient, *T_Ws_* = *d*^2^/*D* (*d* is the effective diffusion thickness, and *D* is the effective diffusion coefficient of the ion species).

(4) $$Z_{Ws}=\frac{R_{Ws}\tan h\left({\left(j\omega T_{Ws}\right)}^{n_{Ws}}\right)}{{\left(j\omega T_{Ws}\right)}^{n_{Ws}}}$$

N values for the Warburg element in Table 1 were 0.27 and 0.24 for Samples D12 and D25, respectively. These values represent the finite length of diffusion in the micro-/nanostructured coating with a transmissive boundary. Furthermore, deviation from the 45° line in the complex plane suggested diffusion in two or three dimensions. Such diffusion paths were also observed in porous media [43]. After functionalization, the EIS spectrum of the D12 sample could best fit with another CPE–resistance-element combination, separati46ng the micro-/nanosurface structure (including possible air gaps) from the nonuniform barrier properties of the Ni film. Nevertheless, the obtained n value was only 0.57, and this CPE–resistance-element combination could be potentially modeled with a Warburg element. According to Figure 5b and results in Table 1, the impedance value obtained for the electrodeposited Ni film on Sample D25 was almost three times larger than that on Sample D12. The different ratio of fine and coarse features in the topography of these two surfaces is responsible for this difference in the impedance values of the two samples. In this regard, *r* as surface-roughness parameter could be utilized to describe the ratio of the sample that comes into contact with the corrosive electrolyte as below:

(5) $$r=\frac{real\;surface\;area}{apparent\;surface\;area}$$

As discussed in Section 3.2.2, the surface of the electrodeposited nickel film on the D25 substrate appeared to be rougher than that formed on Sample D12, thus exposing a larger surface area to electrolytes in comparison with the electrodeposited Ni film on Sample D12. Therefore, qualitatively, the *r* parameter for functionalized film on Sample D25 was greater than that of the functionalized film on Sample D12, which explains the former’s better corrosion resistance (by 1.2 times) than that of the latter.

As described earlier, when the electrodeposited Ni films were functionalized with SA, air pockets formed between sample surface and electrolyte that limited the access of aggressive electrolyte to the sample surface. Consequently, electron transfer decreased [7], which, in turn, increased the corrosion resistance of the functionalized samples. Due to the different ratio of fine and coarse features in the microstructure of the films formed on substrates with different grain sizes, the number of air pockets that were formed on the functionalized Ni film on Sample D12 was smaller than that formed on Sample D25. Therefore, properties of the surface/electrolyte on functionalized film on Sample D25 were governed by the large fraction of the air pockets, changing the equivalent circuit model that described its EIS data, compared to that used for functionalized film on Sample D12.

To verify the reliability of EIS data and their corresponding quantified parameters, we performed PDP measurements on the electrodeposited Ni films on Samples D12 and D25 before and after functionalization. Results from the PDP measurements after 30 min immersion of samples in 3.5 wt % NaCl solution are provided in Figure 5c. We estimated corrosion current density (*i_corr_*) and corrosion potential (*E_corr_*) using the Tafel extrapolation method, and report the corresponding values in Table 2, where apparent surface area (*A*) was used in the calculations. In this table, the corrosion efficiency of the inhibitor (*%η*), which is calculated via the following equation, is also provided.

(6) $$\%\eta =\frac{i_{corr}^{0}-i_{corr}}{i_{corr}^{0}}\times 100$$

where *i*^0^*_corr_* and *i_corr_* are corrosion current densities of films in the absence and presence of SA, respectively [44]. As is evident from the PDP results, the corrosion resistance of Sample D25 was 2.5 times higher than that of Sample D12. After functionalization, corrosion resistance on both samples was comparable, and the functionalized Ni film on Sample D25 showed only slightly better corrosion resistance compared to that formed on Sample D12, due to the larger fraction of air pockets on its surface, the result of which was consistent with EIS results and CA measurements.

## 4. Conclusions

We examined the impact of copper-substrate-grain size on the micro-/nanostructure, hydrophobicity, and corrosion resistance of succeeding electrodeposited nickel films before and after functionalization with a self-assembled stearic acid film. Crystallography and topography analysis revealed that the copper substrate with larger grain size actuated the formation of a hierarchal nanostructure nickel film with preferred growth plane direction of (111) and contact angle lower than 10°, whereas the nickel film, deposited a substrate with a smaller grain size, exhibited a more homogeneous structure, with preferred growth plane direction of (220) and a contact angle of about 56°. After functionalization, deposition of stearic acid film on the samples resulted in a drastic increase in surface hydrophobicity and a contact angle of ~150°. The contact angle of both samples slightly decreased upon exposure to a corrosive medium, but functionalized films maintained their superhydrophobic properties even after 5 days of exposure to a 3.5% NaCl solution. Corrosion resistance of the electrodeposited nickel layer on the copper substrate with the larger grain size was comparatively better than that on the substrate with the smaller grain size. Nevertheless, the corrosion resistance of both samples dramatically increased upon surface functionalization with a self-assembled layer of stearic acid.

## Figures and Tables

**Table 1:** Electrochemical parameters obtained from fitting EIS results in Figure 5a,b using equivalent circuit models in Figure 6.

Sample	*R*_1_	*Q*_1_	*n*_1_	*R*_2_	*R*_Ws_	*T*_Ws_	*n*_Ws_	*Q*_2_	*n*_2_	*R*_3_	*C*_1_	*C*_2_
	(Ω·cm^2^)	(μΩ·cm^−2^S^−*n*^)		(Ω·cm^2^)	(Ω·cm^−2^S^−1/*n*^)			(μΩ·cm^−2^S^−n^)		(Ω·cm^2^)	(μF·cm^−2^)	(μF·cm^−2^)
D12	6.22	86.56	0.92	4180	17,110	30.06	0.27	-	-	-	79.2	-
D25	4.78	98.47	0.94	26,660	56,340	189.7	0.24	-	-	-	104	-
D12 functionalized	6.11	2.07	0.95	25,150	-	-	-	6.56	0.57	260,870	1.75	9.79
D25 functionalized	7.89	5.48	0.88	285,690	-	-	-	-	-	-	5.81	-

**Table 2:** Electrochemical parameters obtained from polarization curves in Figure 5c.

Sample	*E_corr_* (V)	*i_corr_* (A/cm²)	%η
D12	−0.24	1.04 × 10^−6^	-
D25	−0.23	4.26 × 10^−6^	-
Functionalized D12	−0.29	1.78 × 10^−7^	93.08
Functionalized D25	−0.28	1.16 × 10^−7^	82.83
